# Supplementary material for: High social status males experience accelerated epigenetic aging in wild baboons
Source: eLife. 2021 Apr 6;10:e66128. doi: 10.7554/eLife.66128 (PMC8087445; doi:10.7554/eLife.66128)
Supplement: Transparent reporting form [file elife-66128-transrepform.docx]

***eLife’s* transparent reporting form**

We encourage authors to provide detailed information *within their submission* to facilitate the interpretation and replication of experiments. Authors can upload supporting documentation to indicate the use of appropriate reporting guidelines for health-related research (see [EQUATOR Network](http://www.equator-network.org/%20)), life science research (see the [BioSharing Information Resource](https://biosharing.org/)), or the [ARRIVE guidelines](http://www.plosbiology.org/article/info:doi/10.1371/journal.pbio.1000412) for reporting work involving animal research. Where applicable, authors should refer to any relevant reporting standards documents in this form.

If you have any questions, please consult our Journal Policies and/or contact us: [editorial@elifesciences.org](mailto:editorial@elifesciences.org).

**Sample-size estimation**

- You should state whether an appropriate sample size was computed when the study was being designed
- You should state the statistical method of sample size computation and any required assumptions
- If no explicit power analysis was used, you should describe how you decided what sample (replicate) size (number) to use

Please outline where this information can be found within the submission (e.g., sections or figure legends), or explain why this information doesn’t apply to your submission:

We performed a preliminary analysis (using glmnet to estimate epigenetic age) with N = 95 samples to evaluate our statistical power given the sample size and effect sizes. From this preliminary analysis, we chose to analyze >250 samples for the final analysis. The analysis of the full dataset (N=277 samples) is described in the Methods section.

**Replicates**

- You should report how often each experiment was performed
- You should include a definition of biological versus technical replication
- The data obtained should be provided and sufficient information should be provided to indicate the number of independent biological and/or technical replicates
- If you encountered any outliers, you should describe how these were handled
- Criteria for exclusion/inclusion of data should be clearly stated
- High-throughput sequence data should be uploaded before submission, with a private link for reviewers provided (these are available from both GEO and ArrayExpress)

Please outline where this information can be found within the submission (e.g., sections or figure legends), or explain why this information doesn’t apply to your submission:

Samples were collected from 1989-2018 from wild baboons living across the Amboseli basin of southern Kenya as part of a longitudinal series of studies. Biological replicates (N=277) are blood samples from wild baboons; these 277 samples were obtained from 245 animals (13 females and 15 males were each sampled twice, and 1 female and 1 male were each sampled three times). This information is explained in detail in the Methods section. Links to access all sequence data are provided in the Data Availability section.

**Statistical reporting**

- Statistical analysis methods should be described and justified
- Raw data should be presented in figures whenever informative to do so (typically when N per group is less than 10)
- For each experiment, you should identify the statistical tests used, exact values of N, definitions of center, methods of multiple test correction, and dispersion and precision measures (e.g., mean, median, SD, SEM, confidence intervals; and, for the major substantive results, a measure of effect size (e.g., Pearson's r, Cohen's d)
- Report exact p-values wherever possible alongside the summary statistics and 95% confidence intervals. These should be reported for all key questions and not only when the p-value is less than 0.05.

Please outline where this information can be found within the submission (e.g., sections or figure legends), or explain why this information doesn’t apply to your submission:

Exact statistical tests used, and associated information (e.g., N, degrees of freedom, and any methods of multiple test correction) are provided within the main text of the Results section. The full statistical results of linear models are provided as supplementary tables. Raw data points are shown in main figures 1 and 3.

(For large datasets, or papers with a very large number of statistical tests, you may upload a single table file with tests, Ns, etc., with reference to sections in the manuscript.)

**Group allocation**

- Indicate how samples were allocated into experimental groups (in the case of clinical studies, please specify allocation to treatment method); if randomization was used, please also state if restricted randomization was applied
- Indicate if masking was used during group allocation, data collection and/or data analysis

Please outline where this information can be found within the submission (e.g., sections or figure legends), or explain why this information doesn’t apply to your submission:

Experimental groups were not assigned in this study. We targeted animals that were healthy and had not been previously sampled, with focus on animals that were no longer dependent on their mothers (> 4 years old). Sample collection is described in the Methods section, and sample information is provided in Supplementary Table 1.

**Additional data files (“source data”)**

- We encourage you to upload relevant additional data files, such as numerical data that are represented as a graph in a figure, or as a summary table
- Where provided, these should be in the most useful format, and they can be uploaded as “Source data” files linked to a main figure or table
- Include model definition files including the full list of parameters used
- Include code used for data analysis (e.g., R, MatLab)
- Avoid stating that data files are “available upon request”

Please indicate the figures or tables for which source data files have been provided:

Figure 1 (Tables S1); Figure 2 (Table S1); Figure 3 (Table S1)
